# X chromosome escapee genes are involved in ischemic sexual dimorphism through epigenetic modification of inflammatory signals

**DOI:** 10.1186/s12974-021-02120-3

**Published:** 2021-03-12

**Authors:** Shaohua Qi, Abdullah Al Mamun, Conelius Ngwa, Sharmeen Romana, Rodney Ritzel, Arthur P. Arnold, Louise D. McCullough, Fudong Liu

**Affiliations:** 1 Department of Neurology, McGovern Medical School, The University of Texas Health Science Center at Houston, 6431 Fannin Street, Houston, TX 77030 USA; 2 Department of Anesthesiology, Center for Shock, Trauma and Anesthesiology Research (STAR) Center, University of Maryland School of Medicine, Baltimore, MD USA; 3 Department of Integrative Biology and Physiology, UCLA, 610 Charles Young Drive South, Los Angeles, CA 90095 USA

**Keywords:** KDM5C, KDM6A, Histone demethylation, IRF, Inflammation, Microglia, Stroke

## Abstract

**Background:**

Stroke is a sexually dimorphic disease. Previous studies have found that young females are protected against ischemia compared to males, partially due to the protective effect of ovarian hormones, particularly estrogen (E_2_). However, there are also genetic and epigenetic effects of X chromosome dosage that contribute to stroke sensitivity and neuroinflammation after injury, especially in the aged. Genes that escape from X chromosome inactivation (XCI) contribute to sex-specific phenotypes in many disorders. *Kdm5c* and *kdm6a* are X escapee genes that demethylate H3K4me3 and H3K27me3, respectively. We hypothesized that the two demethylases play critical roles in mediating the stroke sensitivity.

**Methods:**

To identify the X escapee genes involved in stroke, we performed RNA-seq in flow-sorted microglia from aged male and female wild type (WT) mice subjected to middle cerebral artery occlusion (MCAO). The expression of these genes (*kdm5c*/*kdm6a)* were confirmed in four core genotypes (FCG) mice and in post-mortem human stroke brains by immunohistochemistry (IHC), Western blot, and RT-PCR. Chromatin immunoprecipitation (ChIP) assays were conducted to detect DNA levels of inflammatory interferon regulatory factor (IRF) 4/5 precipitated by histone H3K4 and H3K27 antibodies. Manipulation of *kdm5c*/*kdm6a* expression with siRNA or lentivirus was performed in microglial culture, to determine downstream pathways and examine the regulatory roles in inflammatory cytokine production.

**Results:**

*Kdm5c and kdm6a* mRNA levels were significantly higher in aged WT female vs. male microglia, and the sex difference also existed in ischemic brains from FCG mice and human stroke patients. The ChIP assay showed the IRF 4/5 had higher binding levels to demethylated H3K4 or H3K27, respectively, in female vs. male ischemic microglia. Knockdown or over expression of *kdm5c*/*kdm6a* with siRNA or lentivirus altered the methylation of H3K4 or H3K27 at the IRF4/5 genes, which in turn, impacted the production of inflammatory cytokines.

**Conclusions:**

The KDM-Histone-IRF pathways are suggested to mediate sex differences in cerebral ischemia. Epigenetic modification of stroke-related genes constitutes an important mechanism underlying the ischemic sexual dimorphism.

**Supplementary Information:**

The online version contains supplementary material available at 10.1186/s12974-021-02120-3.

## Background

Stroke is the leading cause of serious chronic disability and the fifth leading cause of death in the USA [1, 2]. Clinically stroke is a sexually dimorphic disease [3, 4]. Young women have lower stroke incidence than young men thought to be due, in part, to the protective effect of estrogen [5, 6]. After menopause, the female protective stroke phenotype reverses as aged women not only have higher stroke incidence but also higher mortality and worse outcomes than age-matched men [7, 8]. Our previous studies using the Four Core Genotypes (FCG) mouse model have recapitulated this clinical epidemiology. Stroke sensitivity is mediated primarily by gonadal hormones in young animals, regardless of their chromosome complement, but as animals age, chromosomal sex drives ischemic outcomes [9]. Because the number of X chromosomes correlates with susceptibility to ischemic disease, we asked if specific X genes contribute to this phenotype.

It has been recognized for some time that genes on the X chromosome can escape XCI [9]. Some genes escape from XCI during 4–8 cell stage of embryonic development [10], and some others may escape during aging [11, 12], leading to gene dosage imbalance between males and females. As a result, sexually dimorphic phenotypes in normal or disease conditions occur. In humans, about 23% of X-linked genes consistently escape XCI, resulting in sex differences in pathophysiology, behavior, and cognition [13–18]. Among these X-escape genes are *kdm5c* (lysine demethylase 5C) and *kdm6a* (lysine-specific demethylase 6A), which demethylate H3K4me3 and H3K27me3, respectively [19, 20], and are subsequently involved in epigenetic modification of interferon regulatory factor 4 (IRF4) and IRF5 [21]. IRF4 and IRF5 form a regulatory axis that is critical for microglial polarization and immune responses to stroke [22]. Previous studies have shown the expression levels of KDM5C/6A were significantly higher in female vs. male in cardiac tissue after infarction [23]. However, it is unknown if these two X-escapee genes are differentially expressed in the ischemic brain and whether they contribute to stroke sensitivity.

In the present study, we performed MCAO in both wild type (C57BL/6) and FCG mice, a model in which hormonal effects can be dissociated from chromosomal effects [24]. The expression of KDM5C/6A and their downstream IRF signals were examined in these mice. In addition, the expression of *kdm5c*/*6a* was manipulated by siRNA or lentivirus in microglial cultures to determine the effect on downstream signals. In these mechanistic studies, we found that KDM5C/6A is sexual dimorphically expressed in ischemic brain and that these two demethylases regulate the activation of microglia after stroke.

## Methods

### Human brain tissue

All human postmortem brain samples were obtained from the University of Pittsburgh neurodegenerative brain bank, and appropriate ethical permission and informed consent were obtained (Committee for Oversight of Research and Clinical Training Involving Decedents). All cases meeting inclusion criteria in a database search of the University of Pittsburgh brain bank and which had sufficient brain tissue available were included in the study. The database search was performed by the brain bank manager who was not involved in any subsequent analysis. All slides were analyzed by an investigator blinded to the database search case and demographics. Totally 24 male and female (62–92 years, mean = 80.0 years) cases of postmortem brains were used including 6 acute ischemic stroke (1–7 days), and 6 age-matched patients, who died of non-stroke pathology as “controls.” Samples were fixed in formalin and embedded in paraffin. The staining process was performed as previously described [25]. Briefly, after antigen retrieval, the sections were treated with 3% H_2_O_2_ for 10 min at RT to quench endogenous peroxidase and incubated with 3% BSA for 30 min at RT. The sections were then exposed to primary antibodies rabbit anti-KDM5C (1:300; Novus Biologicals) and rabbit anti-KDM6A (1:300; Cell signaling) and mouse anti-Iba-1 (1:300; Fisher scientific) overnight at 4 °C, followed by secondary antibodies, i.e., mouse antibodies labeled with horseradish peroxidase and rabbit antibodies labeled with alkaline phosphatase according to the instructions (ADI-950-100-0001, Enzo). All slides were counterstained with hematoxylin.

### Mice/animals

In FCG mice, males (called XYM here) have a Y chromosome deleted for the testis-determining gene *Sry*, plus an autosomal Sry transgene [26]. XYM mice were bred to C57BL/6J wild-type (WT) females (Jackson Laboratory) to produce FCG mice of four genotypes: XYM/XXM (mice with *Sry*, gonadal males but with different chromosome combination) and XYF/XXF (mice lacking *Sry*, gonadal females but with different X/Y combination) [27]. All mice were housed in an ambient temperature and humidity controlled vivarium, with a 12- to 12-h day night cycle, free access to food and water. All mice were allowed to age to 18–22 months before use. Animal protocols were approved by the University’s Institutional Animal Care and Use Committee and were performed in accordance with the National Institutes of Health and the University of Texas Health Science Center at Houston (UTHealth) animal guidelines.

### Ischemic stroke model

Cerebral ischemia was induced by 60-min reversible MCAO under isoflurane anesthesia as previously described [28]. Rectal temperatures were maintained at approximately 36.5 ± 0.5 °C during surgery with an automated temperature control feedback system. A midline ventral neck incision was made, and unilateral MCAO was performed by inserting a 6.0-mm monofilament (Doccol Corp, Redlands, CA) into the right internal carotid artery 6 mm from the internal carotid/pterygopalatine artery bifurcation via an external carotid artery stump. Reperfusion was performed by withdrawing the suture 60 min after the occlusion. Regional cerebral blood flow was measured in all stroke animals using Laser Doppler flowmetry. Animals that showed a regional cerebral blood flow reduction by at least 85% from baseline levels during MCAO were included for further experimentation. After the MCAO, the mice were administered daily injections of 0.9% sodium chloride, provided with wet mash, and body weight was recorded. Behavioral assessments were performed 72 h after MCAO immediately prior to sacrifice. Sham-operated animals underwent the same surgical procedure, but the suture was not advanced into the middle cerebral artery.

### Western blots

Total protein was extracted from ischemic brains using ice-cold NP-40 cell lysis buffer (50 mM Tris-HCl, pH 7.4, 250 mM NaCl, 5 mM EDTA, 1 mM PMSF, 50 mM NaF, 1 mM Na3VO4, 0.02% NaN3, and 1% NP40) containing freshly added protease inhibitor cocktail (4%). Briefly, protein was mechanically dissociated from tissue using Dounce Tissue Grinders and QSONICA disrupter at 4 °C. The homogenates were centrifuged at 15,000 RPM for 20 min at 4°C, and supernatants were removed and stored in aliquots at −80 °C for downstream experiments. Total protein from brain homogenate samples was quantified by PierceTM BCA Protein Assay kit (Ref # 23225, Thermo Scientific, IL) with protein signals determined at 562 nm, using a PerkinElmer Multimode Plate Reader. Equal amounts of protein (50 μg) were boiled for 5 min and resolved by SDS-PAGE, on pre-cast 4–15% Mini-PROTEAN™ TGX Protein Gels (Bio-Rad Laboratories) at 75–100V for 60 min. The gel was electrophoretically transferred to polyvinylidene fluoride membrane at constant current of 150 mA. Following transfer the membrane was blocked with 5% blocking grade blocker in 1x Tris-buffered saline with Tween (TBS-T, 0.1%) for 1 h, then followed by incubation with primary antibodies: KDM5C (Novus Biologicals; 1:1000) or KDM6A Rabbit mAb (Cell Signaling Technology Inc., 1:1000); Anti-beta Tubulin (Abcam Cambridge; 1:1,000). The blots were further incubated overnight at 4 °C and then washed with TBS-T. The membranes were then incubated with HRP Goat anti-Rabbit IgG antibody (Vector Laboratories, Inc., Burlingame, CA; 1:5,000) for 1 h at room temperature. Blots were visualized using an enhanced chemiluminescence assay kit (Thermo Scientific, IL).

### Brain dissection and immunohistochemistry

Immunohistochemical staining of fixed-frozen sections was performed as described previously [29]. Briefly, the mice were anesthetized by tribromoethanol (Avertin®) IP injection and then transcardially perfused with 0.1M cold PBS (pH 7.4) followed by 4% paraformaldehyde; the brain was post-fixed for 24 h and placed in cyroprotectant (30% sucrose). The brains were cut into 10-μm free-floating sections on a freezing microtome; the sections were then blocked in 0.1M PBS with 0.25% Triton X-100 (Sigma) and 10% donkey serum for 2 h and incubated overnight at 4 °C with the following primary antibodies: Goat anti-Iba-1 (1:300; Novus Biologicals), Mouse anti-GFAP (1:200; Millipore), Mouse anti-NeuN (1:250; Millipore), rabbit anti-KDM5C (1:300; Novus Biologicals), and rabbit anti-KDM6A (1:300; Cell signaling). After washing in TBS + 0.05% Tween 20, the sections were incubated with the indicated secondary antibodies for 1 h. The following secondary antibodies were used: Donkey anti-mouse IgG Alexa Fluor 594 conjugate (1: 500; Invitrogen), donkey anti-rabbit IgG Alexa Fluor 647 conjugate (1: 500; Invitrogen), and donkey anti-goat IgG Alexa Fluor 594 conjugate (1: 500; Invitrogen). The nuclei were stained with DAPI (Invitrogen). Eight 63X fields from each animal were analyzed in the peri-infarct area at the inner boundary zone of the infarct. Double-positive cells were counted by an unbiased, blinded investigator using ImageJ software (NIH), Version 1.52a, and the cell numbers were normalized to sham groups.

### Primary microglia culture

Sex separated, primary cortical microglial cultures were prepared from C57BL/6 aged (18 months) or neonatal (post-natal days 0–2) WT mice brains as described previously [30]. Briefly, the cortices were dissociated via serial incubations with neuronal dissociation kit according to the manufacturer’s instruction (MACS Miltenyi Biotech). The cells were cultured in Dulbecco’s modified Eagle’s media supplemented with 10% fatal bovine serum and 1% penicillin/streptomycin for 10 days at 37 °C and 5% CO_2_. The culture was boosted by replacing L929-conditioned media at days 3 and 5. After 10 days, flasks were shaken for 3 h at 300 × rpm to collect loosely attached microglia. The collected microglia were further cultured in 8-well chamber slides for immunocytochemistry (ICC) and in 6-well plate coated with poly-d-lysine (0.001%, Sigma) for gene expression analysis. The purity of these microglial cultures was 99% as determined by Iba-1 immunoreactivity.

### Oxygen-glucose deprivation (OGD) and siRNA/lentivirus transfection

To model ischemia/reperfusion conditions in vitro, the microglia cultures were exposed to OGD as described previously [30, 31]. The cultured microglia were firstly treated with siRNA or lentivirus specific to *kdm5c* or *kdm6a* (Lentifect™ Lentivirus System; GeneCopoeia). The culture medium was replaced with serum-free, glucose-free Locke’s buffer (154 mM NaCl, 5.6 mM KCl, 2.3 mM CaCl2, 1 mM MgCl2, 3.6 mM NaHCO_3_, 5 mM HEPES, and 5 mg/ml gentamicin, pH 7.2), and the cultures were incubated in an experimental hypoxia chamber in a saturated atmosphere of 95% N2 and 5% CO_2_ for 8 h. After 8 h of OGD, the culture medium were reperfused by replacing normal levels of glucose and were incubated in a humidified atmosphere of 5% CO_2_ incubator for another 24 h before the downstream analysis.

### ChIP analysis

ChIP assays were performed with ChIP-IT High Sensitivity® kit (Active Motif; CA). Briefly, microglial cells (4~5 million) isolated from aged male and female mice were cultured under normoxia or OGD/reperfusion treatment conditions; then, cells were harvested and fixed with fixation buffer (containing 1% formaldehyde). Cross-linked cells were washed with cold PBS 3 times and then resuspended in ChIP buffer supplemented with PIC (Protease Inhibitor Cocktail; Active Motif; CA) and PMSF (Phenylmethylsulfonyl Fluoride; Active Motif; CA). Next, the cells were homogenized and the chromatin was sheared to 100~500bp fragments by sonication. A 500-uL volume (100 ug chromatin mixture) was used per immunoprecipitation, and 5 μL (1% of total) was kept as the input DNA. ChIP was carried out with 5 μL of antibodies (anti-H3K4me1, -H3K4me3, -H3K27me1, -H3K27me3; Active Motif; CA) and IgG (Santa Cruz; CA).

### Quantitative real-time PCR

Immunoprecipitated DNA was quantified by real-time polymerase chain reaction with primer pairs, and each sample was run in triplicate of 384 well plate within CFX384 Touch Real-Time PCR Detection System (Manager Version 3.1.1517.0823.). The primer pairs used were listed in Table 1. ChIP-quantitative PCR (qPCR) data were normalized to the input DNA. The chromatin mixture with IgG antibody was used as a negative control. Each experiment was independently performed in triplicate.

**Table 1:** Primer sequence used

Primers	Sequence
*GAPDH*	5’-GTGTTCCTACCCCCAATGTGT-3’ 5’-ATTGTCATACCAGGAAATGAGCTT-3’
*irf1*	5’-CATGCCAATCACTCGAATGC-3’ 5’-CCTGCTCTCTTAACGTTCACTC-3’
*irf3*	5’-GTGTCACAGCTGGACCTG-3’ 5’-ACCTGGAAGATGCCAAAGTC-3’
*irf4*	5’-AATGGGAAACTCCGACAGTG-3’ 5’-TCACGATTGTAGTCCTGCTTG-3’
*irf5*	5’-GTTTGGTCTGGGTTTTGAGTC-3’ 5’-ATGTCTGTAACCCTAGCACTTG-3’
*irf8*	5’-TGACTTTCCTGCCCATTCC-3’ 5’-GCCCTTTATAGCTGATCCCTG-3’
*IL4*	5’-AACGAGGTCACAGGAGAAGG-3’ 5’-TCCTCGCCACACTTCTCTTT-3’
*CD206*	5’-CCTTTCAGTCCTTTGCAAGC-3’ 5’-CAAGGAAGGTTGGCATTTGT-3’
*Arg1*	5’-CTGAAAGGAGCCCTGTCTTG-3’ 5’-TCACCTGAGCTTTGATGTCG-3’
*TNFα*	5’-CTCCAGCTGGAAGACTCCTCCCAG -3’ 5’-GATCTCAAAGACAACCAACATGTG-3’
*iNOS*	5’-AAGGCCAAACACAGCATACC-3’ 5’-CAAGCACCTTGGAAGAGGAG-3’
*MHCII*	5’-AGCCCCATCACTGTGGAGT-3’ 5’-GATGCCGCTCAACATCTTGC-3’
*Kdm5c*	5′-ACCCACCTGGCAAAAACATTGG-3′ 5′-ACTGTCGAAGGGGGATGCTGTG-3′
*Kdm6a*	5′-CCAATCCCCGCAGAGCTTACCT-3′ 5′-TTG CTCGGAGCTGTTCCAAGTG-3′

### Fluorescence-activated cell sorting (FACS)

Mice were euthanized by tribromoethanol (Avertin ip injection at a dose of 0.25 mg/g body weight), transcardially perfused with 60-mL cold, sterile PBS. After the brains were harvested, the brainstem, cerebellum, and olfactory bulbs were removed. The brain was then divided along the interhemispheric fissure into two hemispheres. Ipsilateral brains were placed in complete Roswell Park Memorial Institute (RPMI) 1640 (Lonza) medium, followed by mechanical and enzymatical digestion with 150 uL collagenase/dispase (1 mg/mL) and 300 uL DNAse (10 mg/mL; both Roche Diagnostics) for 45 min at 37 °C with mild agitation. The cell suspension was filtered through a 70-um filter. Leukocytes were harvested from the interphase of a 70%/30% Percoll gradient. Cells were washed and blocked with mouse Fc Block (eBioscience) prior to staining with the following 4 antibody-conjugated fluorophores: CD45-eF450, CD11b-APC-Cy7, Ly6C-PerCP-Cy5.5, and Ly6G-AF488 for 20 min. For all of the cell surface markers, 0.25 ug (1:100) of antibody was used to stain 1 x 10^6^ cells. All the antibodies were commercially purchased from eBioscience. For live/dead discrimination, a fixable viability dye, carboxylic acid succinimidyl ester (CASE-AF350; Invitrogen), was diluted at 1:300 from a working stock of 0.3 mg/mL. Microglia sorting was performed with BD FACSMelody™ Cell Sorter.

### Statistical analyses

Data from individual experiments were presented as mean ± SD and assessed by Student’s *t* test, one-way ANOVA or two-way ANOVA with Tukey post hoc test for multiple comparisons (GraphPad Prism Software Inc, San Diego, CA,). For two-way ANOVA, significant differences between paired comparisons were conducted with the Holm–Sidak test, whereas the group analysis was compared using Tukey’s post hoc correction. Significance was set at *p* < 0.05.

## Results

### KDM5C and KDM6A expressions are sexually dimorphic in mice microglia

**Fig. 1:**
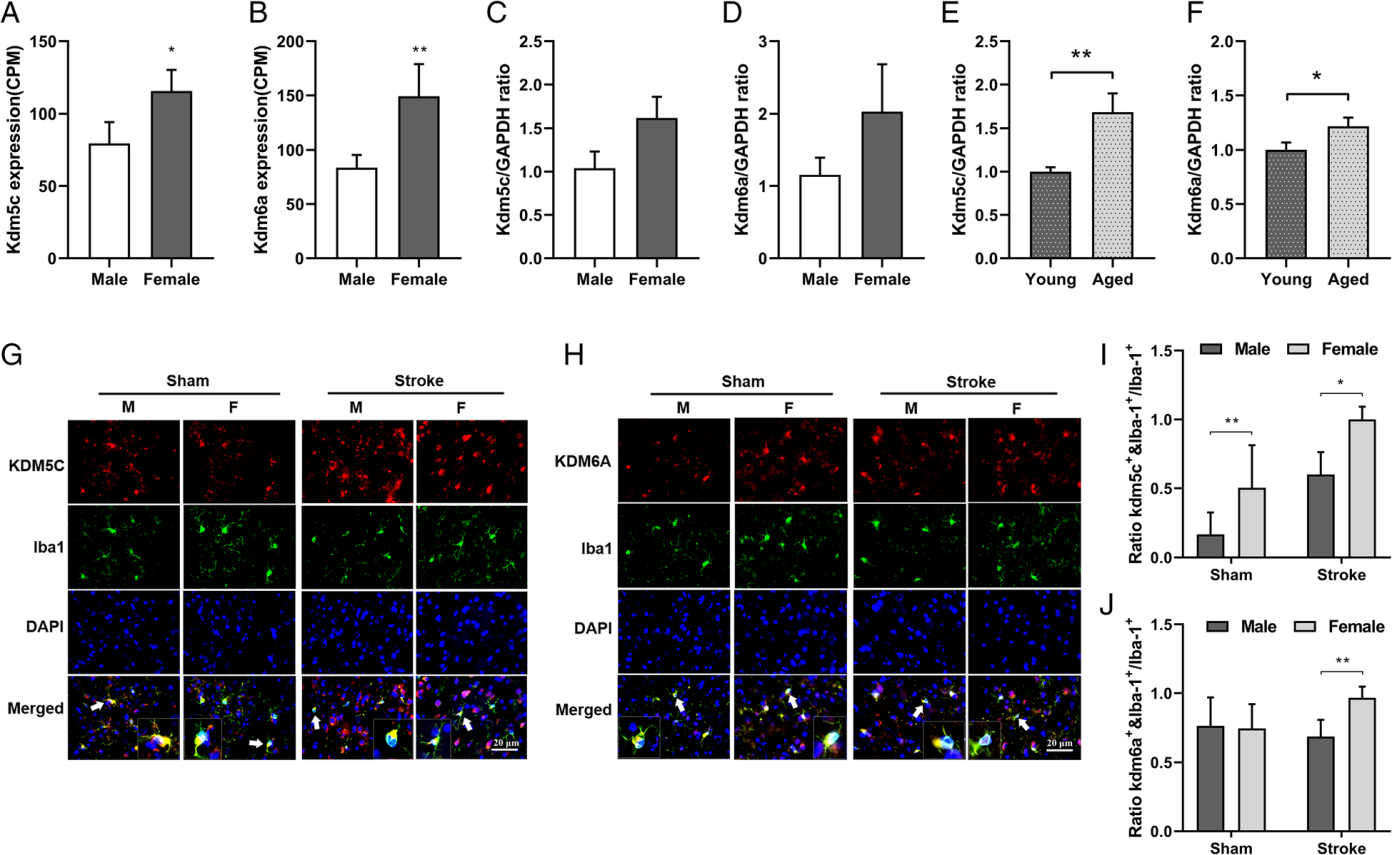
KDM5C and KDM6A expression is sexually dimorphic in microglia. **a**, **b** Flow cytometry sorted microglia were subjected to RNA sequencing, and the results showed aged (18–22 months) female microglia express higher levels of *Kdm5c* and *Kdm6a* compared to male microglia. **c–d** *Kdm5c* and *kdm6a* mRNA levels were measured by RT-QPCR in flow-sorted microglia from young (8–12 weeks) male and female mice, and there was no significant sex difference. **e**, **f** The mRNA levels of *kdm5c* and *kdm6a* were quantified by RT-QPCR in flow-sorted microglia cells from young and aged female mice, and the results showed aged female microglia express significantly higher levels of *Kdm5c* and *Kdm6a* compared to young female microglia. **g**, **h** 63x microscopic fields demonstrate the co-localization of KDM5C/KDM6A and Iba-1; arrows indicate cells with colocalized signals enlarged in the inserts. **i**, **j** Quantification of the ratios of KDM5C^+^/KDM6A^+^&Iba-1^+^ cells over total Iba-1^+^ cells. *n*=5 animals/group; *p < 0.05; **p < 0.01 female versus male mice. Scale bar = 20 μm

The X escapee genes *kdm5c* and *kdm6a* are involved in the epigenetic modification of inflammatory mediators [32, 33] and are expressed in a sexually dimorphic manner in cardiac tissue [23]. To determine whether there is also the sex difference in mice brain resident immune cells (i.e., microglia), a RNA-seq assay was conducted in sorted microglia from aged male and female WT mice and mRNA levels of the two X-escapee genes were analyzed (the volcano plot is in Sup. Fig. 1). Microglia from aged females had significantly higher levels of *kdm5c* compared to male microglia (Fig. 1a). *kdm6a* mRNA also showed the same pattern (Fig. 1b). We also examined mRNA levels of both *kdms* with RT-PCR in sorted microglia from young (8–12 weeks) male and female mice; interestingly, the levels were not significantly increased in young female vs. young male microglia, although females exhibited increasing trends (Fig. 1c, d). When we compared the mRNA levels in sorted microglia in aged female vs. young female mice, we found aged female microglia had significantly higher levels of both *kdms* than their young counterpart (Fig. 1e, f). Taken together, these data suggested that the escape of *kdm5c* and *kdm6a* from XCI is age-dependent [34].

To confirm the RNA-seq findings, we performed IHC in the ischemic brain from aged mice and detected the co-localization of KDMs with Iba-1. The ratio of KDM&Iba-1 double-positive cells over total microglia (Iba-1^+^) was quantified. The ratio of KDM5C^+^&Iba-1^+^ over Iba-1^+^ cells was significantly higher in the sham female vs. male brains, and the same pattern was seen in the stroke group (Fig. 1g, i). We also found that the ratio of KDM6A^+^Iba-1^+^ cells in female stroke brains was significantly higher than that of males (Fig. 1h, j). These data indicate that female microglia express more KDM5C/6A proteins than male microglia after stroke. We also examined neuronal and astrocytic expression of KDMs with IHC and found that astrocytes do not express KDMs (Sup. Fig. 2A&B) and that no sex differences were present in the neuronal expression of either KDM5C or KDM6A in sham or stroke mice (Sup. Fig. 3A-D). These data suggest that the two X-linked genes escape XCI in a tissue- or cell-specific manner.

### Human stroke brains show sex differences in microglial KDM5C and KDM6A expression

**Fig. 2:**
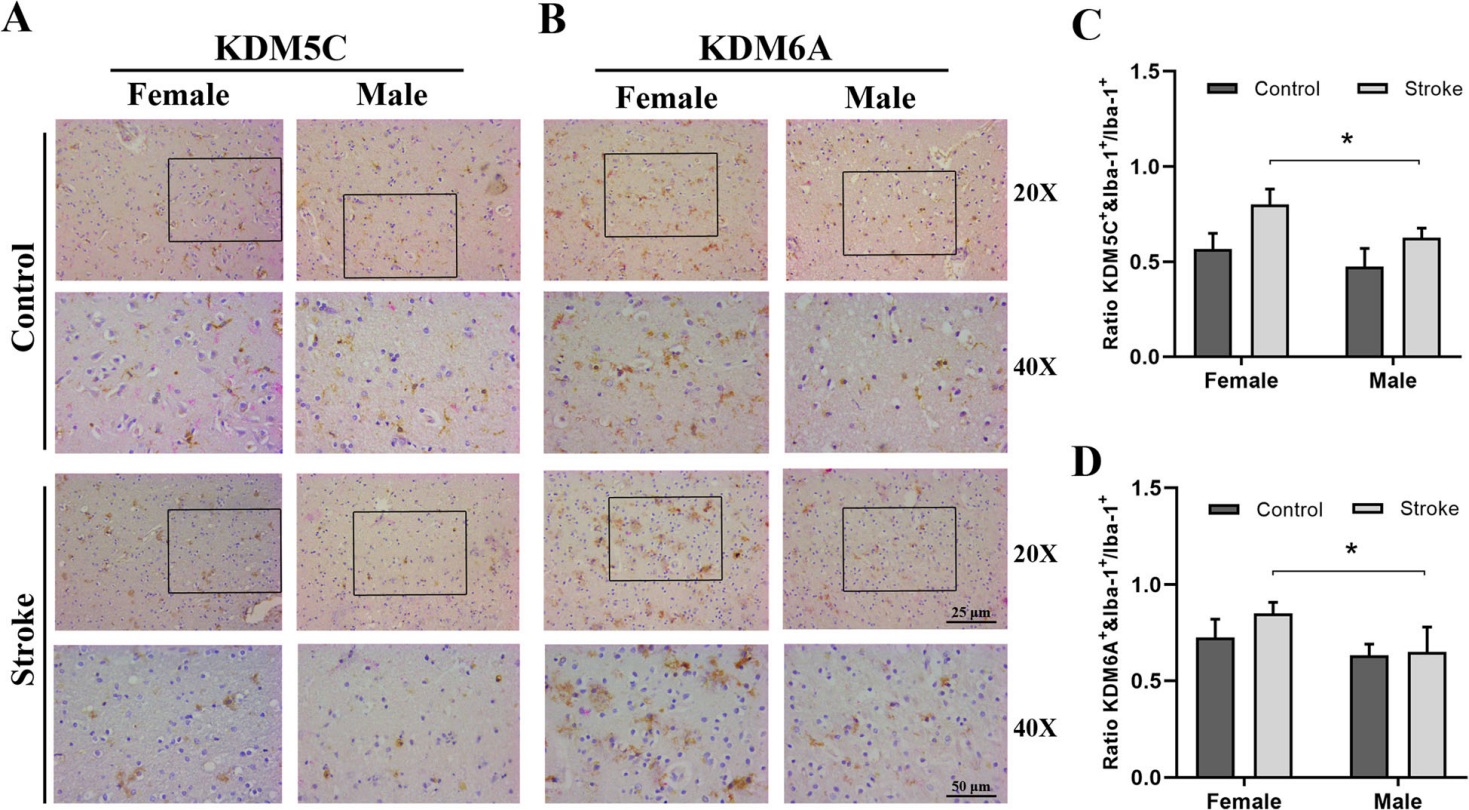
Microglia from aged female stroke patients express more KDM5C and KDM6A than male ischemic microglia. Representative immunohistochemistry microphotographs depicting KDM5C/KDM6A and Iba-1 staining of postmortem human brain tissue from acute ischemic stroke subjects and age-matched control (> 60 years). **a**, **c** 20X and 40X fields (boxed areas in 20X images) demonstrate the co-localization of KDM5C/KDM6A and Iba-1in microglia; scale bar = 25 μm and 50 μm, respectively. **b**, **d** Quantification of the ratios of KDM5C^+^/KDM6A^+^&Iba-1^+^ cells over total Iba-1^+^ cells (*N* = 6 control and 6 stroke subjects/group). *P<0.05

The localization and abundance of either KDM5C or KDM6A and Iba-1 were evaluated by IHC. A ×20 or ×40 magnification images of Iba-1/KDM/hematoxylin-stained sections demonstrated that KDM5C and 6A were expressed extensively (bright red), and the color turned to golden yellow when co-localized with Iba-1 (Fig. 2a, b). The ratio of KDM5C^+^ or KDM6A^+^ and Iba-1^+^ double-positive cell number over Iba-1^+^ cell number was quantified. Although only an increase trend in the ratio was found in female vs. male control brains, the female stroke brains had significantly higher ratio than the male counterparts (Fig. 2c, d), indicating the ischemic stimuli amplified the sex difference in microglial expression of KDM5C/6A. Of note, the IHC performed in human stroke brains can only provide semi-quantitative data, which may not fully reflect the sex difference in microglial expression of the two escapee genes as we only saw the increase trend in control samples. However, the significant sex difference in stroke samples suggested that KDM5C/6A gene escaping from XCI also exists in human microglia, and the ischemic insult amplifies the sex difference.

### Mice with two copies of X chromosome have higher brain expression of KDM6A and KDM5C

**Fig. 3:**
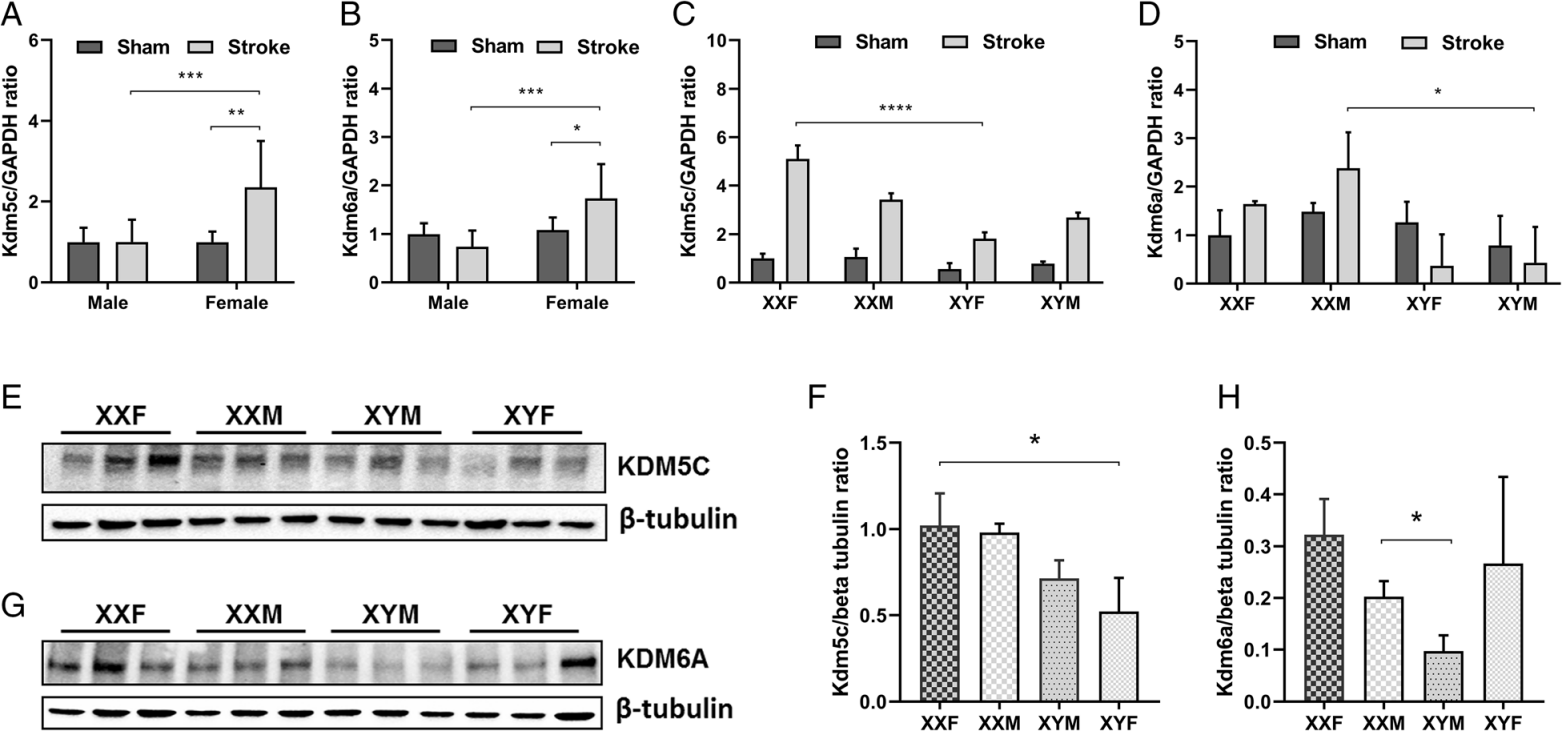
Mice with two copies of the X chromosome have higher expression of KDM6A and KDM5C in ischemic brains. **a**, **b** *Kdm5c* and *Kdm6a* mRNA levels were measured by RT-PCR in aged WT mice brains. **c**, **d** *Kdm5c* and *Kdm6a* mRNA levels in FCG mice brains. **e**, **g** Western blots of KDM5C and KDM6A protein levels in brain homogenates of FCG mice. Tubulin was used as a loading control. **f**, **h** Optical density of KDM5C or KDM6A bands normalized to the loading control. Data were averaged from 3 to 4 independent experiments. *n*=5–6 animals/group, *p < 0.05; **p < 0.01; ***p < 0.001

To examine if the sex difference in microglial KDM expression affect the whole brain KDM levels, we first performed RT-PCR to examine the mRNA levels of *kdm5c* and *kdm6a* in both sham and stroke WT aged mice. Interestingly, the mRNA levels of both *kdm5c* and *kdm6a* were equivalent between male and female sham mice; however, the levels in females significantly increased 3 days after stroke and were significantly higher than their male counterparts (Fig. 3a, b). To confirm this finding, we further examined *kdm5c*/*kdm6a* mRNA levels in brains of aged FCG mice in which comparisons can be done between mice with different sex chromosome combinations. FCG mice have four genotypes: XXF/XYF (gonadal females with either XX or XY chromosome complement) and XXM/XYM (gonadal males with XX or XY sex chromosome complement) [27]. Consistent with the result from WT mice, no differences were seen in sham mice; however, in the stroke cohorts, female mice with two copies of the X chromosome (XXF) had higher *kdm5c* mRNA levels compared to females with one X chromosome (XYF) (Fig. 3c). For *kdm6a*, XXM mice had higher mRNA levels than XYM mice (Fig. 3d). We also examined protein levels of KDMs in FCG mice by western blot after 3 days of MCAO. The KDM5C protein levels were significantly higher in XXF vs. XYF (Fig. 3e, f) mice, and the KDM6A levels significantly higher in XXM vs. XYM mice (Fig. 3g, h), consistent with the mRNA level data.

Taken together, these data suggest that KDM sex specificity in ischemic microglia is so predominant that it even causes a similar sex different pattern in the whole ischemic brain tissue, although the expression of KDMs in the brain exhibits tissue/cell specificity.

### Sex differences in IRF expression after ischemia

**Fig. 4:**
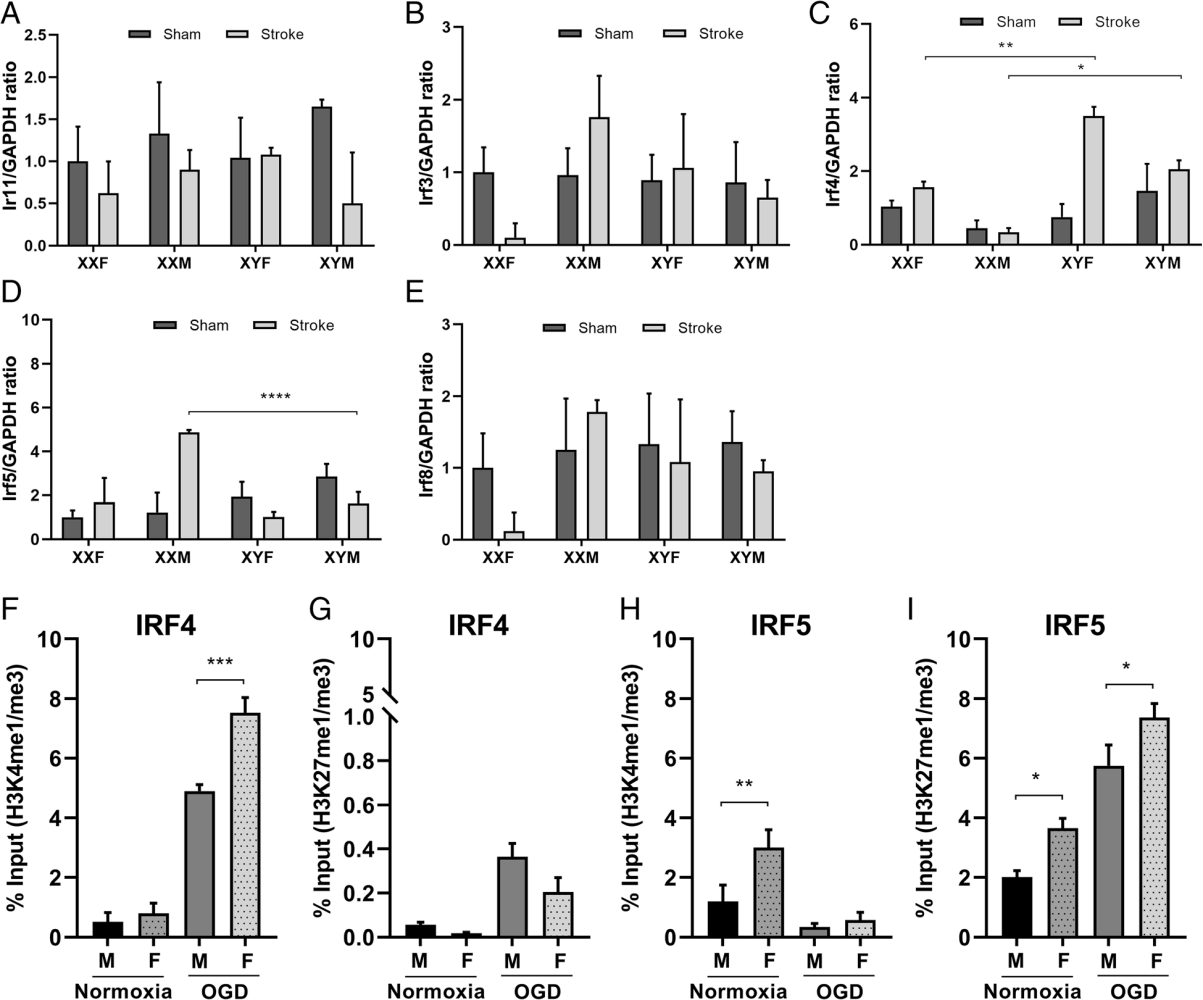
Sex differences in IRF expression after ischemia. **a**–**e** *irf1*, *3*, *4*, *5*, *and 8* gene mRNA levels were measured by RT-PCR in FCG mice brains. Data were averaged from 3–4 independent experiments. *n*=5-6 mice/group. **f–i** The ratio of *irf4/5* gene DNA bound by histone H3K4-me1 over H3K4-me3 and H3K27-me1 over H3K27-me3 form measured by ChIP assays; the raw data are in supplementary Fig. 4. Data were averaged from 3 to 4 independent experiments with triplicate wells. *p < 0.05; **p < 0.01; ***p < 0.001

KDM5C and KDM6A are histone demethylases of H3K4me3 and H3K27me3, respectively [19, 35]. H3K27me3 is repressive to gene expression, but its demethylated form H3K27me1 is active [36]. In contrast, H3K4me3 is active and its demethylated form H3K4me1 is suppressive [37]. These histones epigenetically modify the gene expression of IRFs [38, 39]. As downstream proteins of KDM signaling, it is logical that the expression of IRFs may also exhibit sex differences. To test the hypothesis, we performed RT-PCR to examine mRNA levels of anti-inflammatory (*irf*3, *irf*4) and pro-inflammatory *irf*s (*irf*1, *irf*5, *irf*8) [40] in aged FCG mice brains 3 days after MCAO. Among all the *irf*s, only *irf*4 and *irf*5 mRNA levels showed sex differences in ischemic brains (but not in sham brains), and we did not find any significant difference in *irf*1, 3 and 8 among genotypes of FCG mice (Fig. 4a, b, and e). *irf*4 mRNA levels were significantly higher in XYF vs. XXF and XYM vs. XXM mice (Fig. 4c); however, *irf*5 mRNA levels had a two-fold increase in XXM vs. XYM mice (Fig. 4d). The two *irf*s showed contrasting patterns in mRNA levels as the mice with two copies of X chromosome had higher *irf*5 but lower I*irf*4 than the mice with one copy of the X chromosome. These data suggest that the *irf4* gene was repressed while the *irf5* gene was activated in aged mice with two X chromosomes.

To elucidate if the sex differences in the expression of IRF4/5 were regulated by histone epigenetic modification, we next turned to primary aged microglia cultures. We performed ChIP to examine the interaction of H3K4/H3K27 with IRF4/5. Microglia were cultured from aged WT male and female mice (18 months) and exposed to OGD, an in vitro ischemia model. ChIP was performed in the cell homogenates 8 h after OGD. H3K4 and H3K27 me1 or me3 forms bind to *irf4/5* 5’ open reading frame (ORF) locus, which were detected by the ChIP assay, followed by RT-PCR with *irf4/irf5* primers (Table 1). The ratio of *irf* bound by H3me1 over -me3 form was quantified to predict the rate of the *irf* gene modification. Under normoxic conditions, the ratio of *irf4* bound by H3K4me1/me3 was equivalently low between male and female microglia; however, the ratio dramatically increased after OGD stimulation in both male or female groups, and females showed a significantly higher ratio than males (Fig. 4f), indicating H3K4 methylation produce a more suppressive effect for *irf4* transcription in female microglia after ischemia. The ratio of *irf4* bound by H3K27me1/me3 was 10-fold lower than H3K4 binding and showed no sex difference with either normoxia or OGD treatment, suggesting H3K27 methylation does not induce sex specific transcription of *irf4* (Fig. 4g). The females showed a higher ratio of irf5 bound by H3K4me1/me3 or H3K27me1/me3 in normoxia condition than the males, but the sex difference disappeared for H3K4me1/me3 and persisted for H3K27me1/me3 binding after OGD (Fig. 4h, i). The data suggested that H3K27 modification plays more important roles in IRF5 sexual dimorphic transcription after ischemia than H3K4 binding. The position of the ChIP-RT-PCR primers at *irf* gene structure, and original data of all RT-PCRs and the IgG control were shown in Sup. Fig. 4.

### Manipulation of KDM5C/6A expression in primary microglia culture by lentivirus and siRNA

**Fig. 5:**
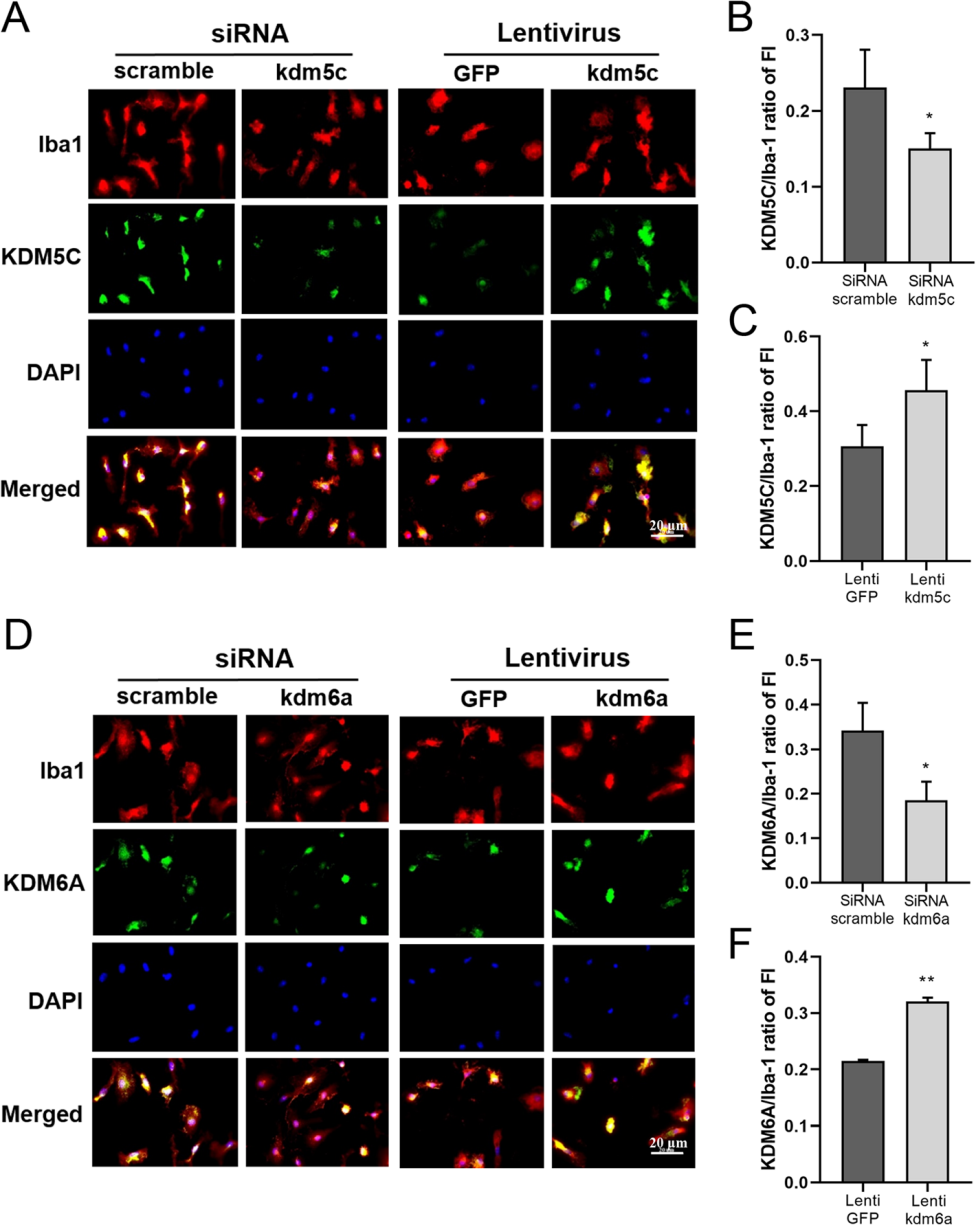
KDM6A/5C expression in primary microglia culture manipulated by lentivirus and siRNA. **a**, **d** ICC staining of microglia with antibodies for KDM5C/KDM6A (green), Iba-1 (red), and DAPI (blue) after *Kdm5c*/*Kdm6a* siRNA and lentivirus treatment. **b**, **c**, **e,** and **f** Ratio of KDM5C/KDM6A over Iba-1 fluorescence intensity (FI). Data were averaged from 21 to 24 microscopic fields from 3 independent experiments. Scale bar = 20 μm (63x). *p < 0.05; **p < 0.01 vs. control groups

Since KDM6A and KDM5C demethylate H3K27me3 and H3K4me3, respectively, and these histones modify *irf* gene (Fig. 4), next we sought to manipulate KDM expression to mechanistically study the KDM-Histone-IRF pathways with neonatal microglial cultures. KDM siRNA or lenti-KDM virus was applied to sex mixed microglia culture to knockdown or overexpress *kdm5c* or *kdm6a*. To validate the effect of the siRNA and lentivirus, ICC was performed to detect the colocalization of KDM5C/KDM6A with Iba-1. As shown in Fig. 5a–c, *Kdm5c* siRNA treatment led to a significant decrease in *Kdm5c* expression, and lenti-*Kdm5c* induced higher expression of *Kdm5c*, compared to their control groups. Similar results were found with *Kdm6a* siRNA and lenti-*kdm6a* treatment (Fig. 5d–f). We further tested mRNA levels of *Kdm5c*/*Kdm6a* with RT-PCR in sex separated microglial cultures after siRNA or lentivirus treatment, and the manipulation had the same effects on the mRNA levels as on the protein expression (Sup. Fig. 5).

### Manipulation of KDM impacts on IRF expression

**Fig. 6:**
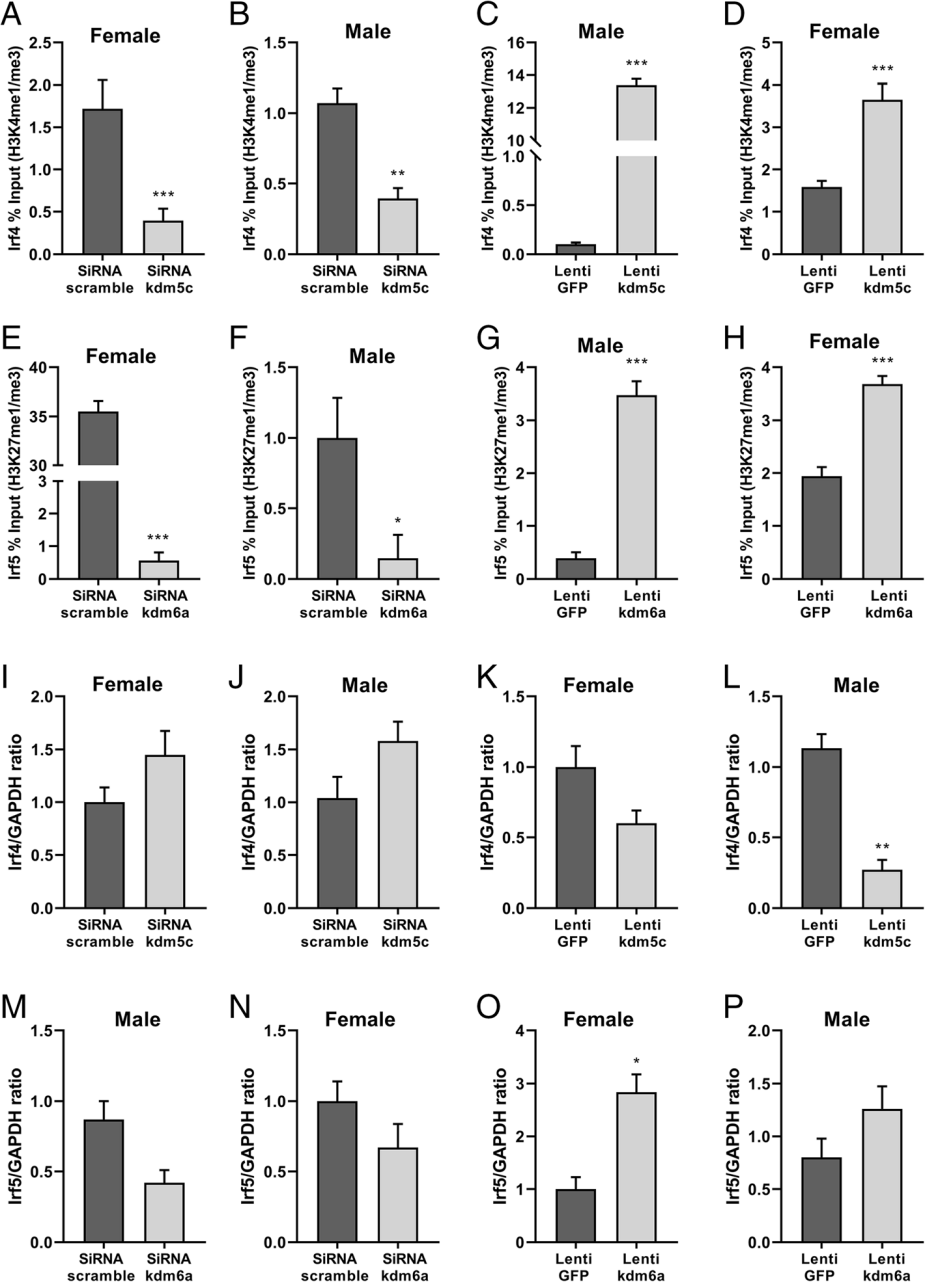
Manipulation of KDM impacts IRF expression in neonatal microglia cultures. **a–d** ChIP analyses show the binding ratio of *irf4* DNA with H3K4-me1 over H3K4-me3 after KDM5C siRNA or lenti-KDM5C treatment in both male and female microglia. **e–h** The binding ratio of *irf5* DNA with H3K27-me1 over H3K27-me3 after KDM6A siRNA or lenti-KDM6A treatment by the ChIP assay. **i**–**l** *irf4* mRNA levels were measured by RT-PCR after KDM5C siRNA or lenti-KDM5C treatment. **m**–**p** *irf5* mRNA levels were measured by RT-PCR after KDM6A siRNA or lentivirus treatment. Data were averaged from 3 to 4 independent experiments with triplicate wells. *p < 0.05; **p < 0.01; ***p < 0.001 vs. control groups

Our in vivo experiments have shown *irf4* and *irf5* DNA were targeted by H3K4me1 and K3K27me1, respectively (Fig. 4), and KDM5C/6A demethylate H3K4me3/H3K27me3 [35, 41]. To investigate if KDMs regulate gene expression of IRFs, we performed ChIP assays in sex separated neonatal microglia cultures to examine the effects of manipulation of KDM on histone epigenetic modification of *irf4*/*5*. In both male and female microglia, *kdm5c* siRNA led to a significant decrease in H3K4me1 binding with *irf4*, demonstrated by a decreased ratio of *irf4* DNA association with H3K4me1 over H3K4me3 compared to scrambled siRNA treatment (Fig. 6a, b); however, lenti-*kdm5c* virus induced a significantly higher ratio compared to the lenti-GFP control (Fig. 6c, d). Similar patterns were seen in the ratio of *irf5* DNA association with H3K27me1 over H3K27me3 after *kdm6a* siRNA and lenti-*kdm6a* virus treatment (Fig. 6e–h).

We also quantified mRNA levels of *irf4*/*5* in cultured microglia. The *irf4* gene mRNA levels showed an increase trend after the *kdm5c* siRNA treatment in both female and male microglia (Fig. 6i, j). After lenti-*kdm5c* treatment, the mRNA levels of *irf4* decreased dramatically in males but moderately in females (Fig. 6k, l). The *irf5* mRNA levels in both female and male microglia showed a decrease trend after *kdm6a* siRNA treatment (Fig. 6m, n), and lenti*-kdm6a* induced a significant increase of IRF5 in females and an increase trend in males (Fig. 6o, p). The results from Fig. 6i–p are consistent with that of Fig. 6a–h, as H3K4me1 is suppressive and H3K27me1 is active for gene transcription, which cause low and high gene expression, respectively. The data indicated that the manipulation of *kdm5c* or *kdm6a* affects histone epigenetic modification status of *irf4 and irf5,* and impacts on *irf4 and irf5* gene expression levels.

### Effects of KDM6A manipulation on microglial cytokine production

**Fig. 7:**
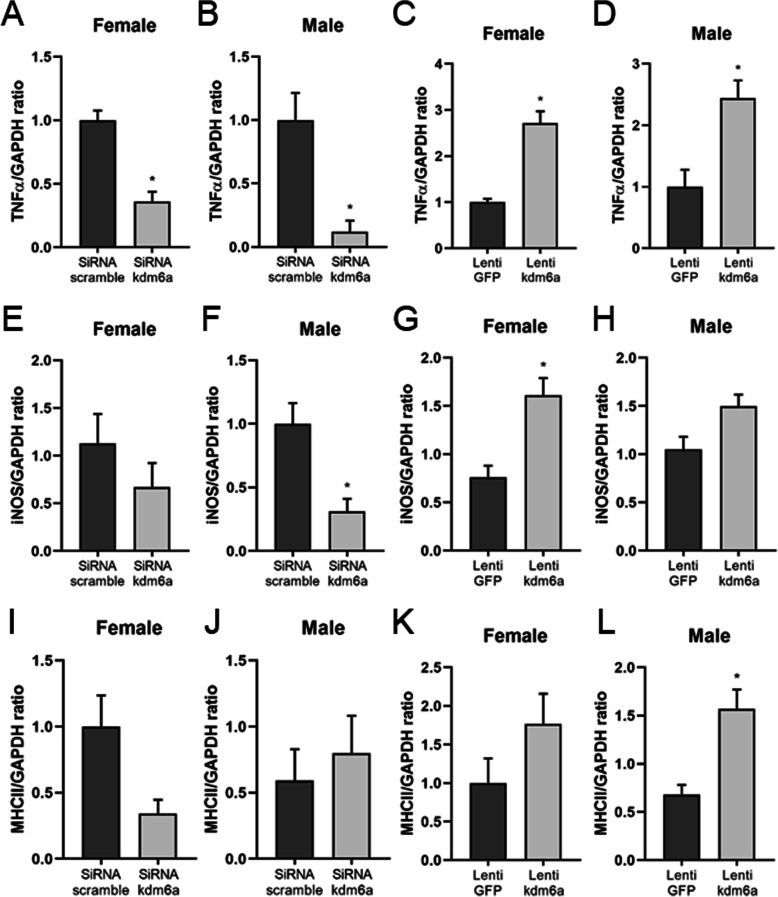
Pro-inflammatory cytokines levels after *Kdm6a* siRNA and lentivirus treatment. **a**–**l** mRNA levels of pro-inflammatory cytokines TNFα, iNOS, and MHCII were measured by RT-PCR in cultured neonatal microglia. Data were averaged from 3 to 4 independent experiments. *n*=5–6 pups/group; *p<0.05

As IRF4/IRF5 regulate gene expression of anti-/pro-inflammatory cytokines respectively in microglia [22], next we examined the levels of cytokines in the homogenates of cultured neonatal microglia by RT-PCR after *kdm5c* or *kdm6a* manipulation. *kdm6a* siRNA treatment led to significant decreases in TNFα mRNA levels in both male and female microglia (Fig. 7a, b), while lenti-*kdm6a* significantly increased TNFα mRNA in microglia of both sexes (Fig. 7c, d). *kdm6a* knockdown by siRNA induced a significant decrease in iNOS mRNA in males with a decrease trend in females, and *kdm6a* overexpression induced a significant increase in pro-inflammatory iNOS in females with an increase trend in males (Fig. 7e–h). Lenti-*kdm6a* caused a significant increase only in male MHCII expression, whereas *kdm6a* siRNA treatment had no effects (Fig. 7i–l). Most of the anti-inflammatory cytokines showed no significant changes (Sup. Fig. 6), with the exception of IL-4, which was significantly increased in female microglia after siRNA treatment (Sup. Fig. 6A) and CD206 that was significantly decreased in males after lenti-*kdm6a* induction (Sup. Fig. 6H). These data indicate that the *kdm6a* signaling mainly regulates microglial pro-inflammatory responses with limited impacts on anti-inflammatory activation.

### Effects of KDM5C manipulation on microglial cytokine production

**Fig. 8:**
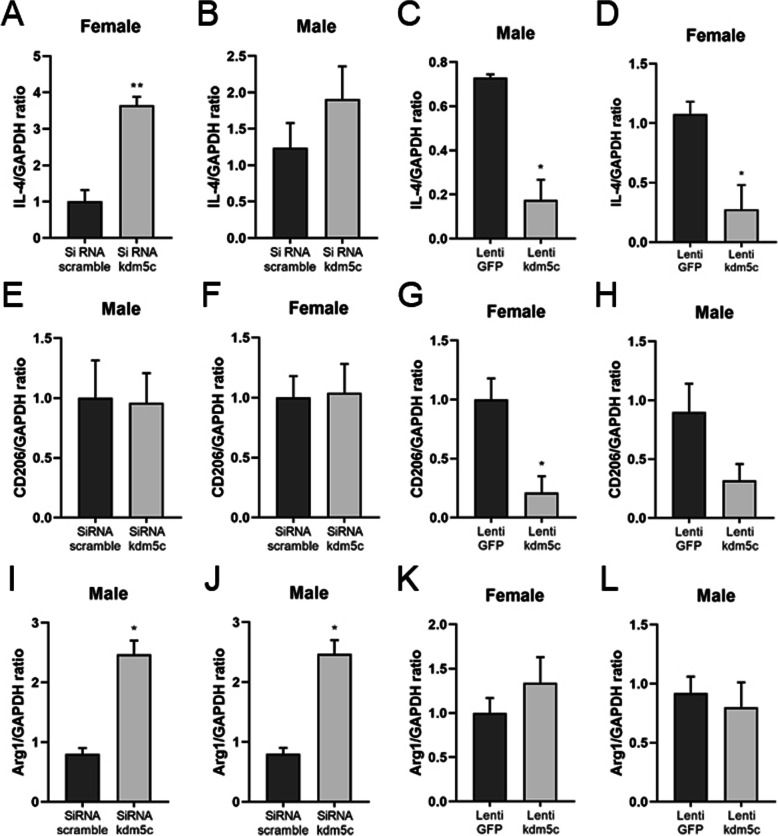
Anti-inflammatory cytokines levels after *Kdm5c* siRNA and lentivirus treatment. **a**–**l** mRNA levels of anti-inflammatory cytokines IL-4, CD206, and Arg1 were measured by RT-PCR in neonatal microglia. Data were averaged from 3 to 4 independent experiments. *n*=5–6 pups/group; *p < 0.05; **p < 0.01 vs. control groups

We also evaluated both pro- and anti-inflammatory cytokine mRNA levels after the manipulation of *kdm5c*. Intriguingly, *kdm5c* manipulation mainly altered the anti-inflammatory response of microglia. IL-4 mRNA levels were significantly affected by both siRNA and lentivirus treatment, and the lentivirus induced significant decreases in IL-4 in microglia of both sexes (Fig. 8a–d). *kdm5c* lentivirus/siRNA also caused significant changes in CD206/Arg1 mRNA levels, respectively, in both male and female microglia (Fig. 8e–l). Most of the pro-inflammatory cytokine mRNA levels showed no significant difference after the *kdm5c* manipulation, with the exception of MHCII that showed a significant decrease in the female group after gene knockdown (Sup. Fig. 7I).

## Discussion

The sex difference in stroke stems from unique signal networks in males or females, which include epigenetic modification of genes that are involved in post-stroke inflammation [42–44]. It has been recently suggested that the epigenetic modification of stroke-related genes can promote post-stroke recovery, whereas aberrant epigenetic modification is associated with the risk of stroke [45–47]. Changes in the epigenome are known to have important consequences to microglial plasticity in the stroke recovery process [48]. In the present study, we investigated two X escapee genes, *kdm5c* and *kdm6a*, which encode histone demethylases and target H3K4 and H3K27 methylation, respectively. We have found KDM5C/KDM6A are sex-specifically expressed in microglia and ischemic brains in both human and mice, although the sex difference has cellular variability. We explored downstream inflammatory pathways influenced by activation of these two demethylases, i.e., KDM5C associates with *irf4* and KDM6A with *irf5* signaling. In addition, our data suggested that KDM5C-H3K4-IRF4 and KDM6A-H3K27-IRF5 pathways regulate microglial production of inflammatory mediators. Our findings demonstrated that the inflammatory responses of microglia can be altered by the manipulation of these two X escapee genes, suggesting KDM5C/KDM6A contribute to the sex difference in stroke through epigenetic modification, and by virtue of the difference in dose of X genes in males and females.

While hormones are a potent and pervasive force that drive sex differences in stroke in young subjects [49], our recent studies identified that the sex chromosome complement also contributes to stroke sensitivity in aged mice. Microglia from mice with two copies of the X chromosome exhibited a more robust pro-inflammatory phenotype compared to those with one X after ischemia [50]. Sex differences in microglia have been well documented in the literature [51–53], and data from our previous and present studies suggest that it is a driving force for stroke sensitivity in males and females. Microglial activation is a fundamental pathophysiological process that mediates post-stroke neuroinflammation and plays an initiating and perpetuating role in the immune response [54]. Post-stroke inflammation causes secondary neuronal damage [55]; therefore, it is critical to control microglial activation as it can shape the immune response and subsequently the extent of the ischemic injury. The present study focused on sex-specific pathways underlying microglial activation and sheds new lights on sex differences in stroke.

Our data demonstrated that aged female microglia have significantly higher expression of KDM5C and KDM6A than their male counterparts, and this is not surprising as *kdm5c/6a* are genes that escape XCI. X-escapee genes are referred to those genes that are not silenced during X-inactivation but are expressed from both X chromosomes [56]. With aging, XCI becomes more unstable and some genes escape XCI resulting in overexpression in females vs. males, which contributes to several diseases [57, 58], which was also reflected by our data (Fig. 1c–f). Several XCI escapee genes are more highly expressed in mice with two X chromosomes than those with one X chromosome in a variety of tissues, including *kdm5c/6a* [42, 59]. Surprisingly, although we found the two genes are differently expressed in aged female vs. male microglia, the sex difference did not translate to the whole brain tissue as WT and FCG sham mice of the two sexes have equivalent expression regardless of the X and Y chromosome complement. This could be due to the reason that tissue variability exists in XCI or gene escaping. Extensive cellular heterogeneity of XCIs has been reported [60], and the escape from XCI varies in murine tissues [61]. Our findings are consistent with these previous reports as sex differences in KDM5C/6A were seen in microglia but not in neurons (Sup. Fig. 2B). Intriguingly, the sex difference in both KDMs can be seen in the whole brain tissue after stroke although not in sham brains (Fig. 3a, b), for unknown reasons. It is likely that the baseline sex difference in microglial expression of *kdm6a*/*kdm5c* is masked by their global expression in the brain; however, these XCI escapees in microglia may be susceptible to the ischemic insult, and once a stroke occurs, the baseline sex difference is amplified leading to a global sex difference in the aged ischemic brains. The susceptibility to stroke was also seen in human microglial expression of the two escapees (Fig. 2), although the semi-quantitative data provide limited information.

KDM5C and KDM6a are histone demethylases of H3K4me3 and H3K27me3, respectively [19, 20, 62]. During brain development and maturation, both KDM5C and KDM6A are ubiquitously expressed and participate in the process of neuronal development and differentiation [63, 64]. Mutation of KDM5C is frequently detected in X-linked intellectual disability (XLID) patients [65], and its escape from XCI is an intrinsic feature of the KDM5C locus [66]. KDM5C functions to balance H3K4 tri-, di-, and mono methylation at the promoter and enhancer locus and therefore keeps an accurate control of target gene expression [19]. KDM6A is also known as UTX (ubiquitously transcribed tetratricopeptide repeat, X chromosome) and catalyzes the demethylation of tri/dimethylated histone H3K27 [67]. Demethylation of H3K4me3 and H3K27me3 by KDM5C and KDM6A to their me1 forms confers repressive and activational effect on gene expression, respectively [19, 68]. Expression of the two X escapee genes from both X-alleles should lead to more robust demethylation and transcriptional effect in females vs. males. Excitingly, our data have indeed confirmed these effects: H3K4me1 and H3K27me1 bind to more IRF4/5 DNA, respectively, in female vs. male microglia (Fig. 4f, i) after OGD; however, mice with two copies of X have higher IRF5 and lower IRF4 level compared to one X mice after stroke (Fig. 4c, d). These delicate regulations of gene expression by KDM5C/6A speak for the importance of studying gene escape in sexually dimorphic diseases. Of note, IRF4 also relates to H3K27me1 (so does IRF5 to H3K4me1); however, the binding levels were relatively low and no sex difference was seen after OGD (Fig. 4g, h).

IRFs are known to play key roles in innate immune responses by regulating microglia/macrophage polarization [22, 69, 70], a function that is tightly controlled by epigenetic modification through DNA hypermethylation and demethylation. Among all IRFs, only IRF4 and IRF5 showed sex-specific expression in aged mice brains after stroke (Fig. 4a–e). IRF4 and IRF5 are two important transcription factors that regulate microglial classical (pro-inflammatory) and alternative activation (anti-inflammatory) respectively. It has been demonstrated by us [22, 70] and others [71, 72] that IRF5 regulates the expression of pro-inflammatory cytokines (TNFα, iNOS, MHCII, etc.), whereas IRF4 mediates the expression of anti-inflammatory cytokines (IL-4, CD206, Arg1, etc.) in microglia or macrophages. The two IRFs may be responsive to many upstream regulatory signals; however, the X escapee genes KDM5C/6A mediate the IRFs’ expression in a sex-biased way through their demethylating functions. By performing in vitro mechanistic studies with siRNA and lentivirus, we have suggested that the KDM5C-H3K4-IRF4 and KDM6A-H3K27-IRF5 regulatory pathways impact on the production of inflammatory cytokines (Figs. 7 and 8). Of note, our data showed that the KDM5C-IRF4 signaling mainly directs anti-inflammatory cytokine production, whereas the KDM6A-IRF5 signaling impacts on pro-inflammatory cytokines primarily (Figs. 7 and 8). The two pathways have limited effects on the other branch of the inflammatory response (i.e., KDM5C on pro- and KDM6A on anti-inflammatory cytokines (Sup. Fig. 6 and 7), suggesting a specific effect of the two X escapee genes in mediating microglial responses. The in vitro assays were conducted to explore the mechanistic pathways downstream to KDM5C/6A, and we did not examine sex differences, as our data showed male and female microglia responded to the manipulation of the two demethylases in a similar way.

## Conclusion

In summary, the present study examined the expression of two X escapee genes (*kdm5c*/*kdm6a*) in microglia and the aged ischemic brains and explored the pathways through which the two demethylases regulate microglial cytokine production. We have revealed that KDM5C/6A tissue-specifically escape from XCI in the brain, which is sensitive to ischemic insults. The present study suggested that the KDM5C-H3K4-IRF4 and KDM6A-H3K27-IRF5 pathways regulate microglial response to ischemia, which however needs to be further confirmed by in vivo assays. We conclude that the epigenetic modification of inflammatory signals by X escapee genes is a novel mechanism in inducing sex differences in stroke and may also apply to other sexually dimorphic diseases.

## Supplementary Information

**Additional file 1: Supplementary Figure 1.** Volcano plot of the RNA-seq showing *kdm5c/6a* gene expression in aged male and female microglia. **Supplementary Figure 2.** KDM6A/KDM5C are not expressed on astrocytes. **Supplementary Figure 3.** No sex difference in KDM6A/5C expression in neurons. **Supplementary Figure 4.** Irf4/5 gene levels with H3K4me1/3 and H3K27me1/3 modification in normoxia and OGD treated aged microglia culture. **Supplementary Figure 5.** KDM6A/5C mRNA levels in lentivirus and siRNA treated microglia. **Supplementary Figure 6.** Anti-inflammatory cytokines levels after *Kdm6a* siRNA and lentivirus treatment. **Supplementary Figure 7.** Pro-inflammatory cytokines levels after *Kdm5c* siRNA and lentivirus treatment.

## Data Availability

The datasets used and/or analyzed during the present study are available from the authors on reasonable request.

## Abbreviations

ChIP: Chromatin immunoprecipitation
FCG: Four Core Genotypes
ICC: Immunocytochemistry
IHC: Immunohistochemistry
IRF4/5: Interferon regulatory factor4/5
KDM5C: Lysine demethylase 5C
KDM6A: Lysine-specific demethylase 6A
MCAO: Middle cerebral artery occlusion
OGD: Oxygen-glucose deprivation
ORF: Open reading frame
TSS: Transcription start site
UTX: Ubiquitously transcribed tetratricopeptide repeat, X chromosome
XCI: X chromosome Inactivation
XLID: X-linked intellectual disability
