# Supplementary material for: X chromosome escapee genes are involved in ischemic sexual dimorphism through epigenetic modification of inflammatory signals
Source: J Neuroinflammation. 2021 Mar 12;18:70. doi: 10.1186/s12974-021-02120-3 (PMC7953638; doi:10.1186/s12974-021-02120-3)
Supplement: Supplementary file 1 — Additional file 1: Supplementary Figure 1. Volcano plot of the RNA-seq showing kdm5c/6a gene expression in aged male and female microglia. Supplementary Figure 2. KDM6A/KDM5C are not expressed on astrocytes. Supplementary Figure 3. No sex difference in KDM6A/5C expression in neurons. Supplementary Figure 4. Irf4/5 gene levels with H3K4me1/3 and H3K27me1/3 modification in normoxia and OGD treated aged microglia culture. Supplementary Figure 5. KDM6A/5C mRNA levels in lentivirus and siRNA treated microglia. Supplementary Figure 6. Anti-inflammatory cytokines levels after Kdm6a siRNA and lentivirus treatment. Supplementary Figure 7. Pro-inflammatory cytokines levels after Kdm5c siRNA and lentivirus treatment. [file 12974_2021_2120_MOESM1_ESM.pdf]

Additional file for

X chromosome escapee genes are involved in ischemic sexual dimorphism through epigenetic modification of inflammatory signals

Shaohua Qi[1, Δ], Abdullah Al Mamun[1, Δ], Conelius Ngwa[1], Sharmeen Romana[1], Rodney Ritzel[2], Arthur P. Arnold[3], Louise D. McCullough[1], Fudong Liu[*, 1]

[1]Department of Neurology, The University of Texas Health Science Center at Houston, McGovern Medical School, 6431 Fannin Street, Houston, TX 77030, US

[2]Department of Anesthesiology, Center for Shock, Trauma and Anesthesiology Research (STAR) Center, University of Maryland School of Medicine, Baltimore, MD, USA

[3]Department of Integrative Biology and Physiology, UCLA, 610 Charles Young Drive South, Los Angeles, CA 90095, USA

[Δ]These authors contribute equally to this work

* Corresponding author: Fudong Liu

Department of Neurology, McGovern Medical School,

University of Texas Health Science Center at Houston, 6431 Fannin Street, Houston, TX 77030, USA.

Email: Fudong.Liu@uth.tmc.edu

Tel/Fax: +1 (713) 500-7038

This PDF file includes:

Supplementary online material:

Supplementary Figure 1: **Volcano plot of the RNA-seq showing *kdm5c/6a* gene expression in aged male and female microglia**.

Supplementary Figure 2: **KDM6A/KDM5C are not expressed on astrocytes**.

Supplementary Figure 3: **No sex difference in KDM6A/5C expression in neurons**.

Supplementary Figure 4: **Irf4/5 gene levels with H3K4me1/3 and H3K27me1/3 modification in normoxia and OGD treated aged microglia culture**.

Supplementary Figure 5: **KDM6A/5C mRNA levels in lentivirus and siRNA treated microglia**.

Supplementary Figure 6: **Anti-inflammatory cytokines levels after *Kdm6a* siRNA and lentivirus treatment**.

Supplementary Figure 7: **Pro-inflammatory cytokines levels after *Kdm5c* siRNA and lentivirus treatment**.

Supplementary Figure 1:

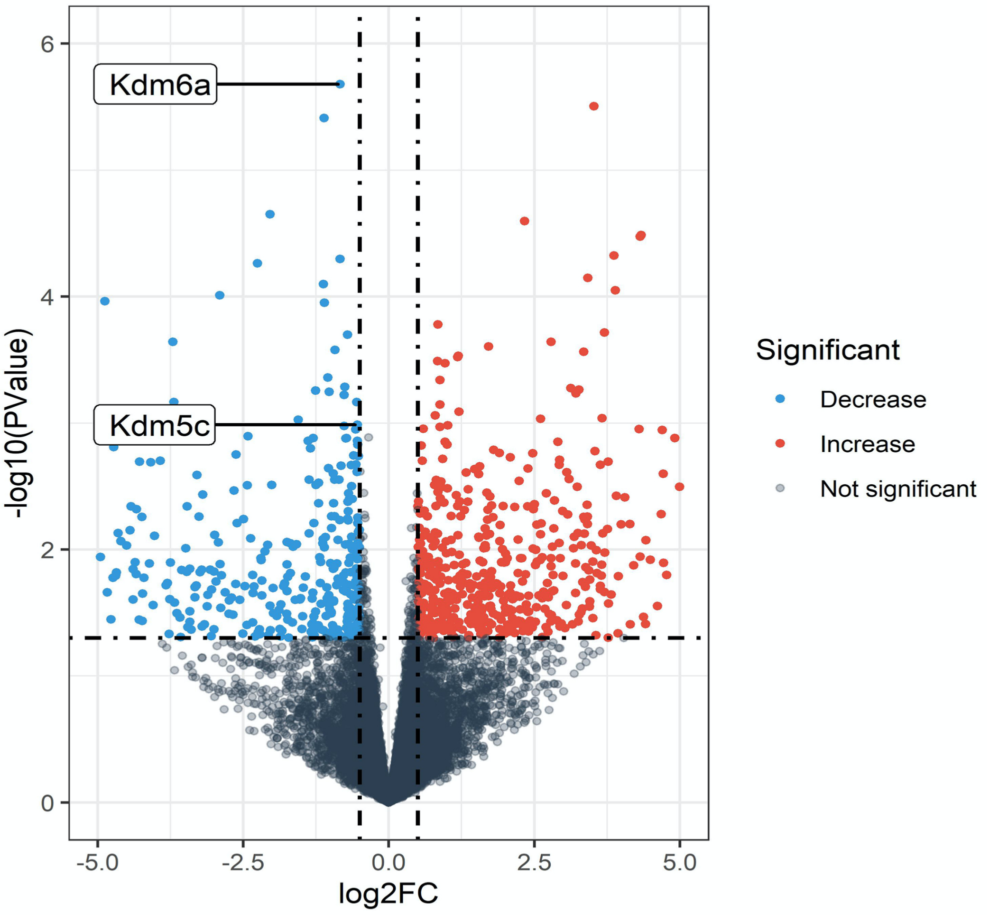

Volcano plot of the RNA-seq showing *kdm5c/6a* gene expression in aged male vs. female microglia. RNA-seq was performed in flow-sorted microglia from aged male and female mice brain tissue. The vertical axis (y-axis) corresponds to the mean expression value of -log10 (P value), and the horizontal axis (x-axis) displays the log2 fold change value. The red dots indicate the RNAs with up-regulated expression (male vs. female), the blue dots indicate the RNAs with down-regulated expression, and the black dots indicate the RNAs with no significant differences between groups. The expression fold change (male/female) >1.5 and with FDR adjusted P < 0.05 are considered statistically significant.

Supplementary Figure 2:

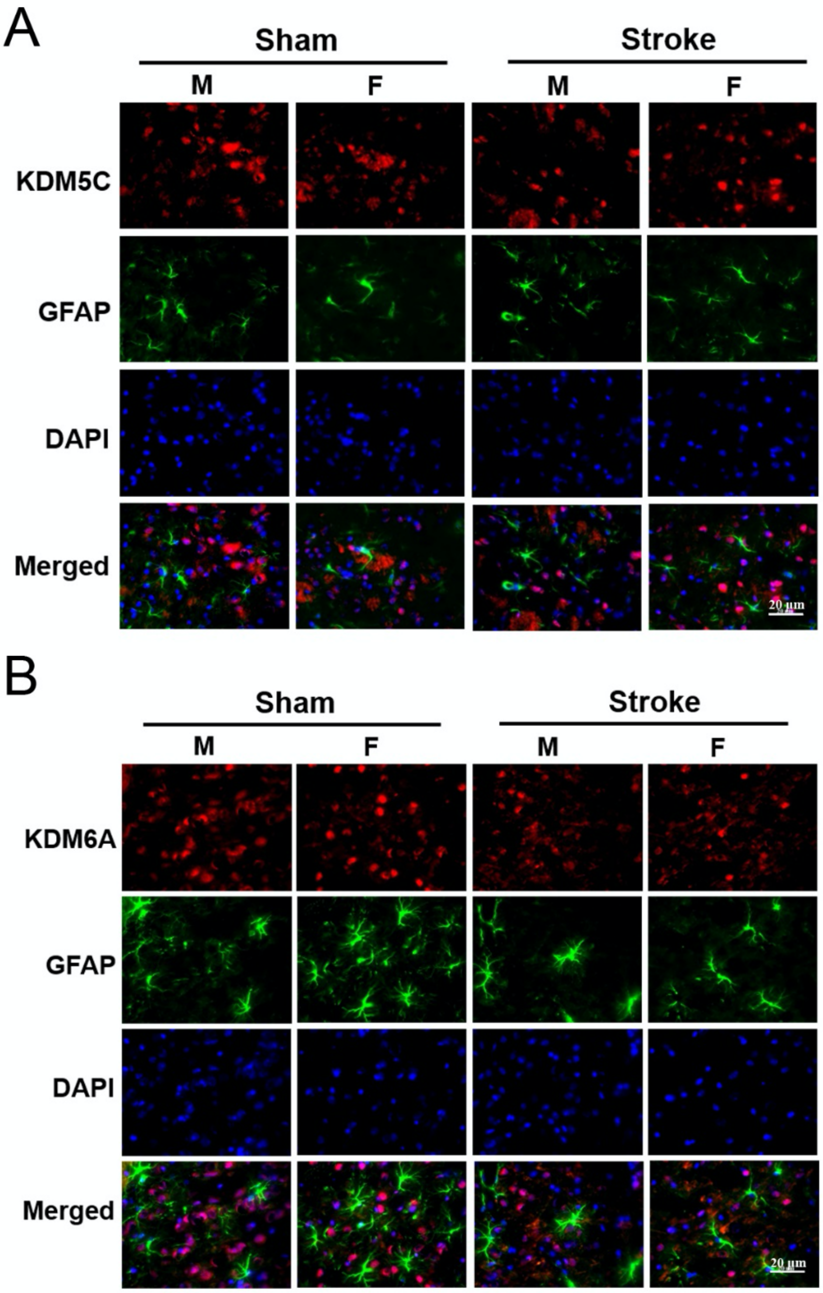

KDM6A/KDM5C are not expressed on astrocytes. (A-B) Peri-infarct area of the WT aged mice brains were stained with KDM5C/KDM6A (red), GFAP (green) and DAPI (blue). Scale bar = 20μm (63x).

Supplementary Figure 3:

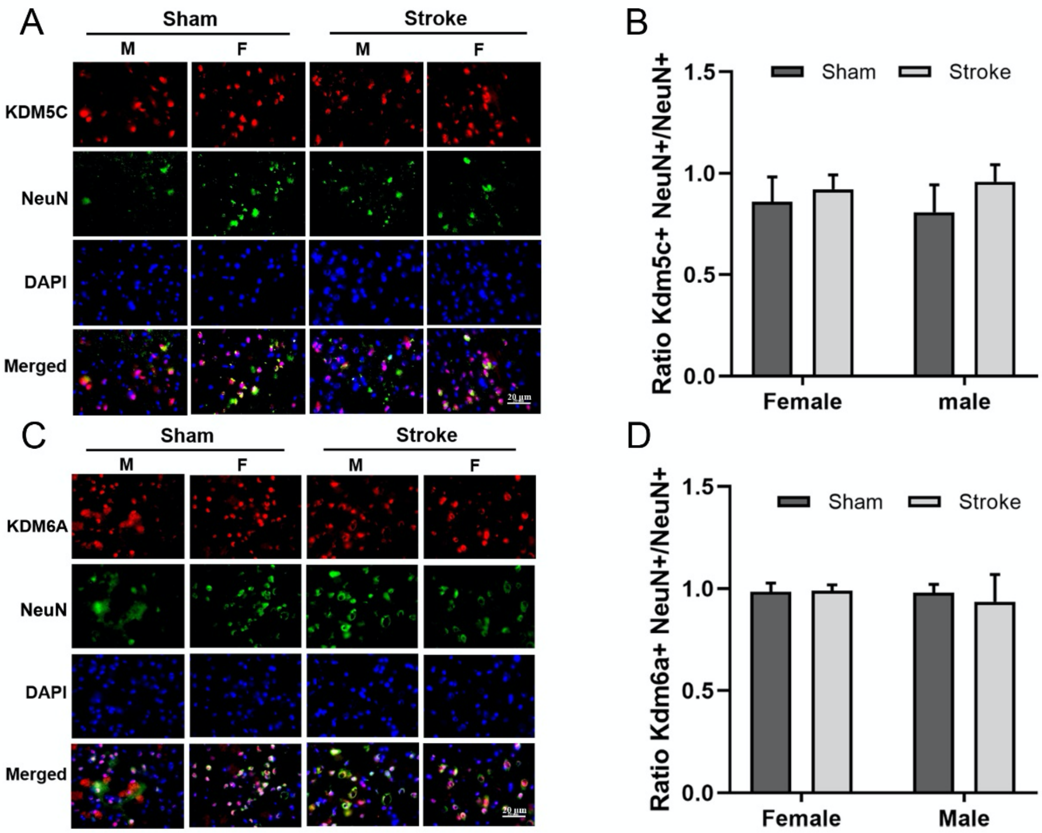

No sex difference in KDM6A/5C expression in neurons. (A and C) Peri-infarct area of the WT aged mice brains were stained with KDM5C/KDM6A (red), NeuN (green) and DAPI (blue). (B and D) Semi-quantification of the ratios of $KDM5C^{+}$/$KDM6A^{+}$& $NeuN^{+}$ cells over total $NeuN^{+}$ cells. n=5 animals/group. Scale bar = 20μm (63x).

Supplementary Figure 4:

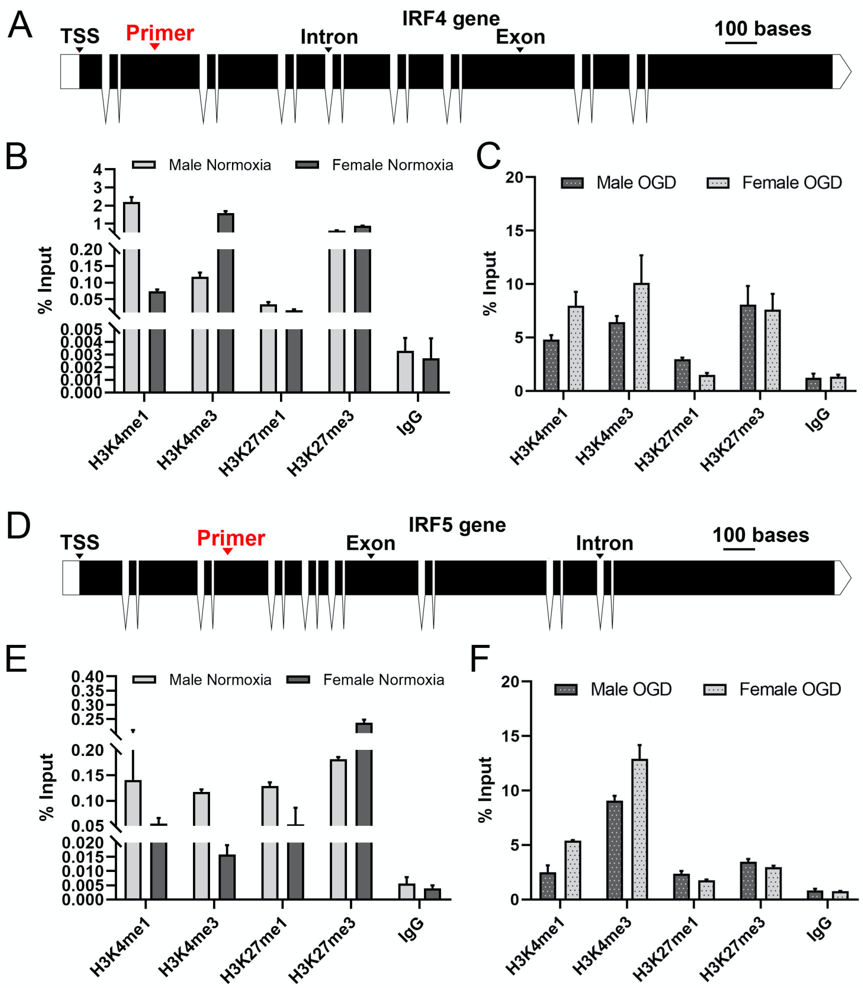

Irf4/5 gene levels with H3K4me1/3 and H3K27me1/3 modification in normoxia and OGD treated aged microglia culture. (A and D) The schematic diagram indicates the structure of *irf4* and *irf5* genes, including the TSS, exons, introns and primer sites. Scale bar = 100 bases. (B-C & E-F) The percentage of IRF4 (B&C) and IRF5 (E&F) DNA levels in the input precipitated by Histone H3K4me1/3 and H3K27me1/3 antibodies, measured by ChIP-RT-PCR at ORF region of *irf4/5* genes in normoxia and OGD treated condition. Microglia were flow-sorted from aged male and female mice. IgG antibody served as a negative control for histone methylation antibodies. Data were averaged from 3-4 independent experiments. n=5-6 mice/group.

Supplementary Figure 5:

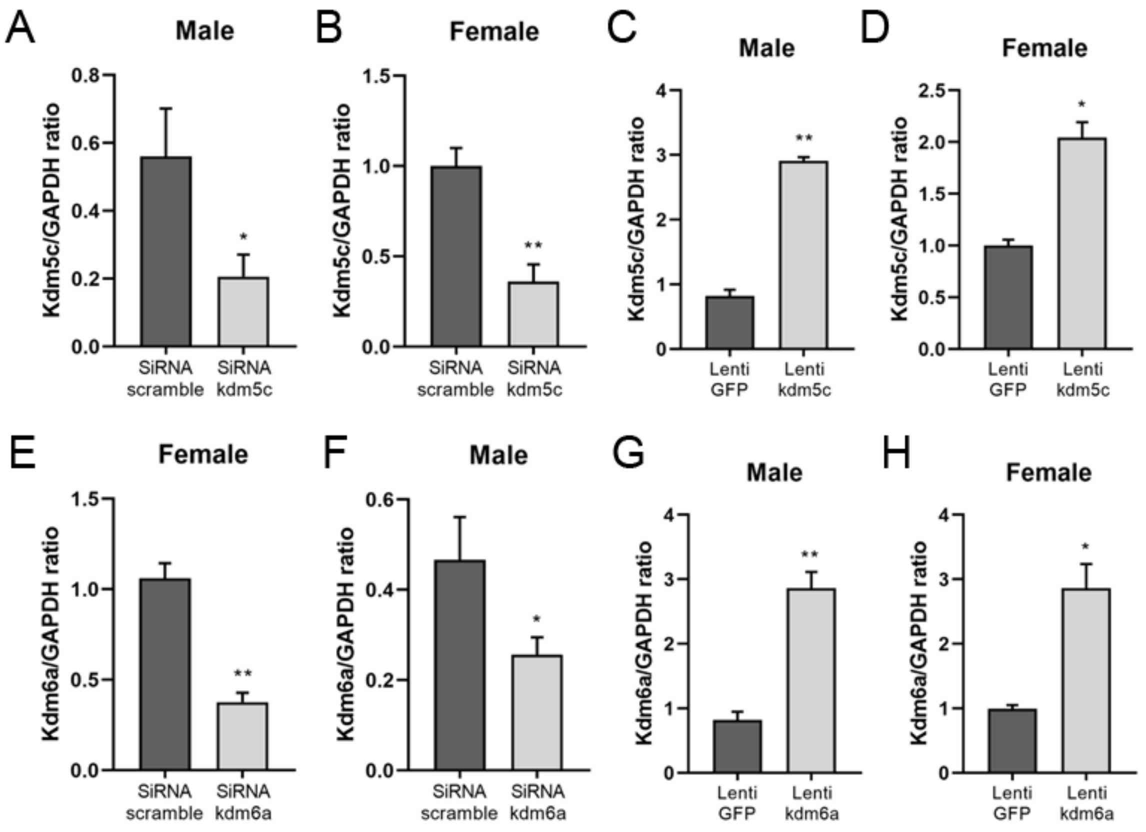

KDM6A/5C mRNA levels in lentivirus and siRNA treated microglia. (A-H) *Kdm5c* and *Kdm6a* gene mRNA levels were measured by RT-PCR in neonatal microglia after KDM5C/KDM6A siRNA or lenti-KDM5C/KDM6A treatment. Data were averaged from 3-4 independent experiments. n=5-6 pups/group; *p < 0.05; **p < 0.01 vs. control groups.

Supplementary Figure 6:

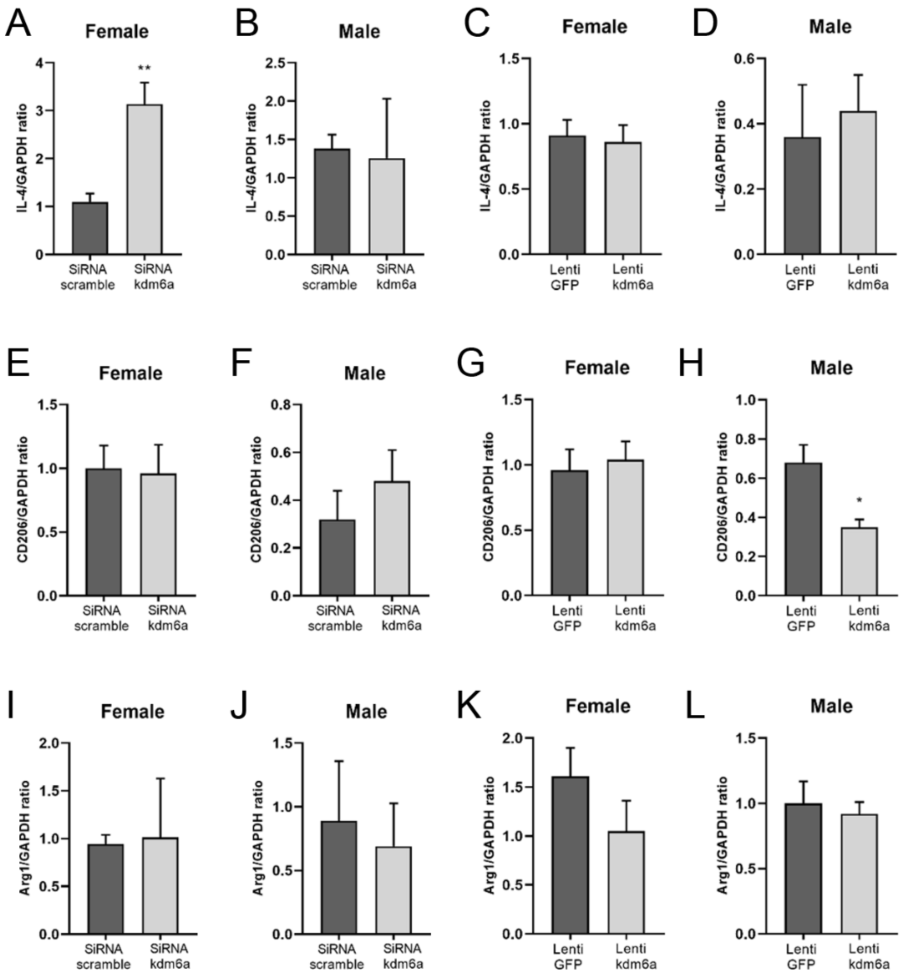

Anti-inflammatory cytokines levels after *Kdm6a* siRNA and lentivirus treatment.

(A-L) Anti-inflammation cytokines IL-4, CD206 and Arg1 gene mRNA levels were measured by RT-PCR in neonatal microglia. Data were averaged from 3-4 independent experiments. n=5-6 pups/group, $^*p < 0.05$; $^{**}p < 0.01$ vs. control groups.

Supplementary Figure 7:

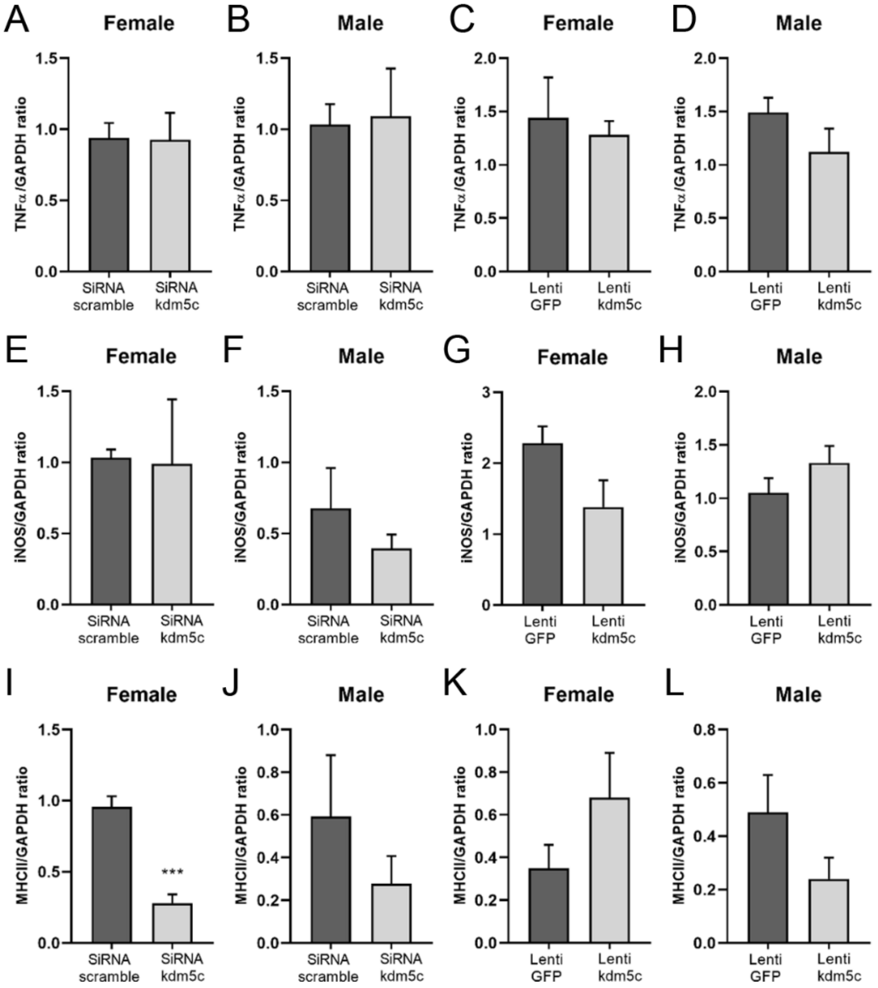

Pro-inflammatory cytokines levels after *Kdm5c* siRNA and lentivirus treatment. (A-L) Pro-inflammation cytokines TNFα, iNOS and MHCII gene mRNA levels were measured by RT-PCR in neonatal microglia. Data were averaged from 3-4 independent experiments. n=5-6 pups/group, ***p < 0.001 vs. control groups.
